# An assessment of false positive rates for malaria rapid diagnostic tests caused by non-*Plasmodium* infectious agents and immunological factors

**DOI:** 10.1371/journal.pone.0197395

**Published:** 2018-05-14

**Authors:** Michelle L. Gatton, Sadmir Ciketic, John W. Barnwell, Qin Cheng, Peter L. Chiodini, Sandra Incardona, David Bell, Jane Cunningham, Iveth J. González

**Affiliations:** 1 School of Public Health and Social Work, Queensland University of Technology, Brisbane, Australia; 2 Malaria Branch, Division of Parasitic Diseases and Malaria, Centers for Disease Control and Prevention, Center for Global Health, Atlanta, Georgia, United States of America; 3 Drug Resistance and Diagnostics, Australian Defence Force Malaria and Infectious Diseases Institute, Brisbane, Australia; 4 Hospital for Tropical Diseases and London School of Hygiene and Tropical Medicine, London, United Kingdom; 5 FIND (Foundation for Innovative New Diagnostics), Geneva, Switzerland; 6 Global Malaria Programme, World Health Organization, Geneva, Switzerland; Université Pierre et Marie Curie, FRANCE

## Abstract

**Background:**

Malaria rapid diagnostic tests (RDTs) can produce false positive (FP) results in patients with human African trypanosomiasis and rheumatoid factor (RF), but specificity against other infectious agents and immunological factors is largely unknown. Low diagnostic specificity caused by cross-reactivity may lead to over-estimates of the number of malaria cases and over-use of antimalarial drugs, at the cost of not diagnosing and treating the true underlying condition.

**Methods:**

Data from the WHO Malaria RDT Product Testing Programme was analysed to assess FP rates of 221 RDTs against four infectious agents (Chagas, dengue, Leishmaniasis and Schistosomiasis) and four immunological factors (anti-nuclear antibody, human anti-mouse antibody (HAMA), RF and rapid plasma regain). Only RDTs with a FP rate against clean negative samples less than 10% were included. Paired t-tests were used to compare product-specific FP rates on clean negative samples and samples containing non-*Plasmodium* infectious agents and immunological factors.

**Results:**

Forty (18%) RDTs showed no FP results against any tested infectious agent or immunological factor. In the remaining RDTs significant and clinically relevant increases in FP rates were observed for samples containing HAMA and RF (*P*<0.001). There were significant correlations between product-matched FP rates for RF and HAMA on all RDT test bands (*P*<0.001), and FP rates for each infectious agent and immunological factor were also correlated between test bands of combination RDTs (*P*≤0.002).

**Conclusions:**

False positive results against non-*Plasmodium* infectious agents and immunological factors does not appear to be a universal property of malaria RDTs. However, since many malaria RDTs have elevated FP rates against HAMA and RF positive samples practitioners may need to consider the possibility of false positive results for malaria in patients with conditions that stimulate HAMA or RF.

## Introduction

People residing in tropical regions of the world are often exposed to a variety of infectious and non-infectious diseases, many of which have similar clinical presentation. Diagnostic tests are available for some conditions, while others need to be diagnosed based on clinical symptoms, after exclusion of other possible causes. Hence available diagnostic tests need to be both sensitive and specific to ensure patients are treated for the true cause of illness. This is particularly important if the disease profile or incidence is changing, as is the case for malaria where the incidence in 2016 was 18% lower globally, and 48% lower in the South-East Asia region, compared to 2010 [1]. Low diagnostic specificity may lead to over-estimation of the number of malaria cases and over-use of antimalarial drugs, at the cost of not diagnosing and treating the underlying condition.

Malaria rapid diagnostic tests (RDTs) are used in many regions of the world, particularly where quality assured microscopy is not available. Malaria RDTs most commonly use *Plasmodium falciparum* histidine-rich protein 2 (PfHRP2) to detect *P*. *falciparum* (Pf) and pan-, Pf-, or *P*. *vivax*-specific *Plasmodium* lactate dehydrogenase (pLDH) to differentiate Pf from non-falciparum and *P*. *vivax* (Pv) infections. Aldolase is also used by some RDTs to detect *Plasmodium spp*. infections. A study assessing cross-reactivity of 10 malaria RDTs with human African trypanosomiasis (HAT) indicated that seven had significantly lower specificity on the pan-pLDH and PfHRP2 RDT test bands against samples from HAT patients compared to paired controls [2]. Reports of false positive (FP) results caused by other infectious agents are limited and usually from small studies. False positive results on one brand of PfHRP2 RDT were reported for patients with acute *Schistosoma mekongi*, but not in chronically infected individuals or individuals with *S*. *japonicum* or *S*. *haematobium*, or on a Pf-pLDH-based RDT [3]. A case report also documents a FP result on the PfHRP2 band of a malaria RDT for a patient with *Salmonella typhi* [4]. Importantly, there does not appear to be an elevated rate of FP in HIV patients with two studies reporting good specificity of selected malaria RDTs in this cohort [5, 6].

In contrast it is well documented that malaria RDTs return FP results in patients with high titres of rheumatoid factor (RF), particularly against PfHRP2-test bands, with product specific FP rates ranging from 2.2–26% [7–10]. However there is a dearth of information about the potential cross-reactivity of malaria RDTs with other immunological factors.

This study presents the results of a secondary analysis of data generated by the WHO Malaria RDT Product Testing programme to investigate the frequency of false positive RDT results against several infectious agents and immunological factors.

## Methods

In this study data from Rounds (Rds) 1 to 6 of the WHO Malaria RDT Product Testing programme was collated to assess the performance of all malaria RDTs submitted for testing against a variety of non-*Plasmodium* infectious agents and immunological factors. The RDT testing methods have been described elsewhere [11]. The specific focus here is the comparison between the product-specific FP rate against clean negative samples obtained from blood banks in malaria endemic and non-endemic settings and FP rates against samples containing non-*Plasmodium* infectious agents and immunological factors. Clean negative samples are defined as samples collected from afebrile patients, with no known infectious disease, blood dyscrasia or immunological abnormality. Samples containing non-*Plasmodium* infectious agents and immunological factors are referred to as ‘dirty negative’ samples and were sourced from diagnostic specimens in the US and Philippines, or from commercial suppliers. All samples, irrespective of their source or classification as clean or dirty negatives, were confirmed to be negative for *Plasmodium* by nested PCR [12]. Samples originating from the Philippines were also screened as negative for malaria by microscopy and RDT (SD Bioline Malaria Ag Pf/Pan RDT, catalogue number 05FK60) at the time of collection. Ethical approval for specimen collection in the Philippines, and transport and archiving for the purpose of product testing was provided by the Research Institute for Tropical Medicine Institutional Review Board and the WHO Ethics Review Committee. Samples originating from the US were purchased from blood banks that collect blood for sale.

The non-*Plasmodium* infectious agents tested included Chagas disease antibody positive plasma (n = 8), dengue antibody positive whole blood and sera (n = 16 for Rds 1–4; n = 24 for Rds 5–6), Leishmaniasis antibody positive sera (n = 20) and Schistosomiasis antibody positive whole blood and sera (n = 24 for Rds 4 and 6; n = 40 for Rds 1–3). No Schistosomiasis samples were tested in Round 5. Each product was also tested against the immunological factors panel: anti-nuclear antibody (ANA) positive sera (n = 52), human anti-mouse antibody (HAMA) positive plasma (n = 12), rheumatoid factor (RF) positive whole blood and sera (n = 16 for Rds 1–5; n = 24 for Rd 6) and rapid plasma regain (RPR) positive sera (n = 20 for Rds 4–5; n = 28 for Rd 6; n = 36 for Rds 1–3). Results from HAMA testing in Round 1 were excluded as only 4 tests were conducted per product. In Rds 5 and 6 commercially produced samples purchased from Seracare (USA) were also used to supplement the ANA, RPR, RF and dengue diagnostic specimens. Sera and plasma samples were reconstituted with packed cells prior to testing on RDTs.

The intensity of each test band on the RDT was graded from 0 to 4 according to a standard colour chart, with 0 representing a negative result and all other intensities classified as positive. As each of the samples was known to be free from *Plasmodium* parasites, any positive test result against an immunological factor or infectious agent was considered to be a FP.

Statistical analysis was conducted using SPSS Version 22 (IBM Corporation). Only results for RDTs which had a FP rate against clean negative samples of less than 10% on the test band/s of interest were included in the analysis, reflecting the threshold used by the World Health Organization (WHO) for procurement [13]. Paired t-tests were used to compare product FP rates on clean negative samples to FP rates on disease-specific samples and samples containing immunological factors. Spearman rank correlation was used to assess correlations between FP rates.

## Results

Data for a total of 235 RDTs was available, however only 221 RDTs met the inclusion criteria for analysis of <10% FP rate against clean negative samples. A total of 40 RDTs (18.1%) did not return any FP results against any of the dirty negative samples containing non-*Plasmodium* infectious agents or immunological factors (that is, they had a FP rate of 0%) (Table 1). Details of these 40 products are listed in S1 Table.

**Table 1:** Summary of RDT characteristics and performance against ‘dirty negative’ samples containing non-*Plasmodium* infectious agents or immunological factors.

RDT Type	No. included in analysis	No. with no FP on any ‘dirty negative’ sample (%)
Pf-only product	63	16 (25.3%)
Pf-pan combination product	97	10 (10.3%)
Pf-Pv combination product	50	9 (18.0%)
Pan-only product	11	5 (45.0%)
Total	221	40 (18.1%)

### False positive results on *P*. *falciparum* test bands

**Fig 1:**
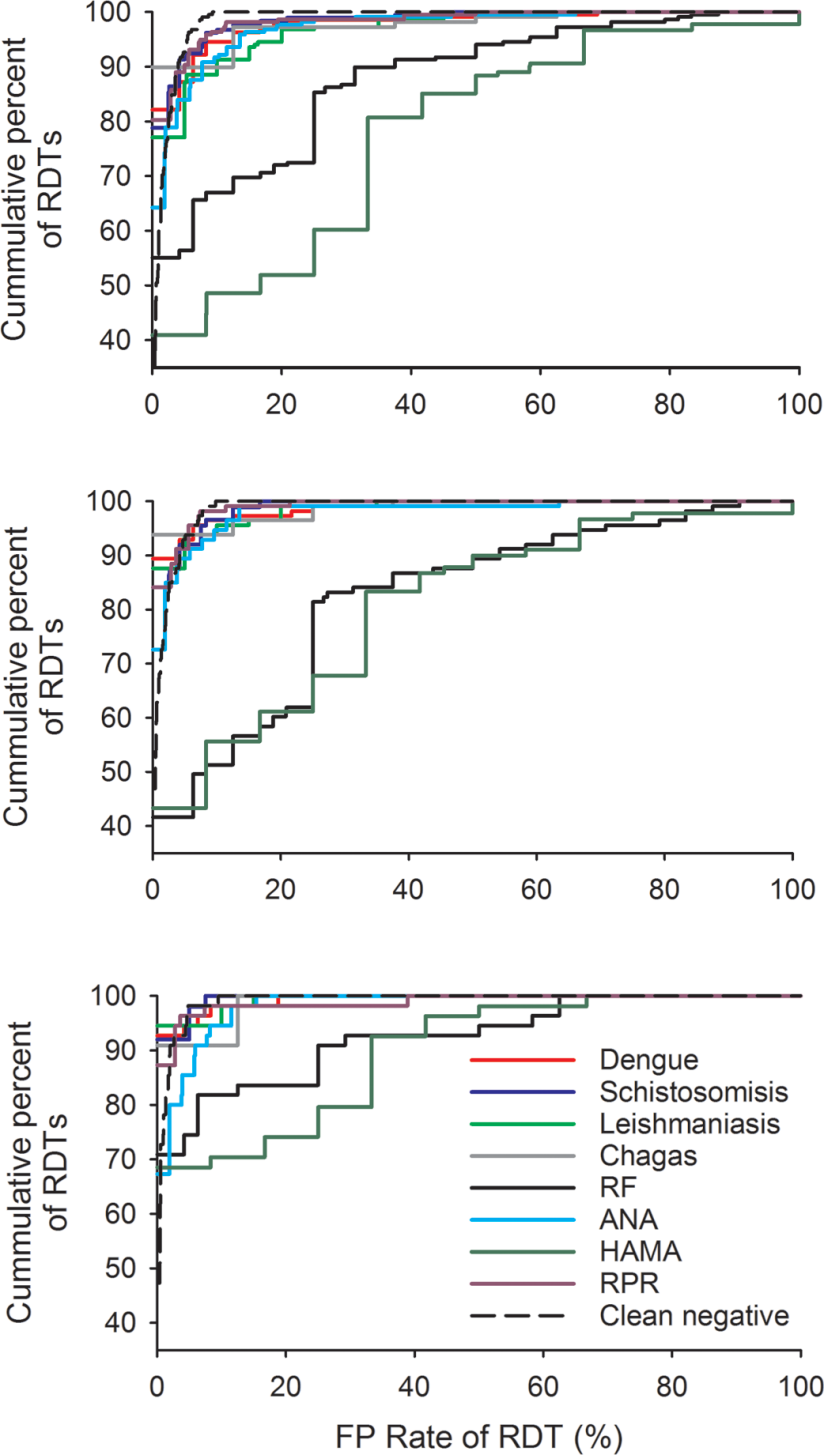
Cumulative distribution of false positive (FP) rates of malaria RDT bands against eight infectious agents and immunological factors. Top panel: Pf-detecting RDT bands; middle panel: pan-detecting RDT bands; bottom panel: Pv-detecting RDT bands.

The large majority (92.4%, 204/218) of *P*. *falciparum* test bands detected PfHRP2, with 17 (8.2%) products detecting Pf-pLDH, either alone or in combination with PfHRP2. Overall the FP rates of *P*. *falciparum* test bands were low, with more than 45% of RDTs having no FP against individual infectious agents or immunological factors, and 20.6% (45/218) having no FP against any of the infectious agents or immunological factors tested (Fig 1). There were no differences between the paired FP rates on the *P*. *falciparum* test band against clean negative samples and samples containing dengue, Schistosomiasis, Chagas, or RPR (*P*>0.10; Table 2). Statistically significant differences in FP rates were detected between clean negative samples and samples containing Leishmaniasis (*P* = 0.002) and ANA (*P* = 0.005), however the differences were small and unlikely to be clinically relevant (Leishmaniasis: mean difference = 1.67%, 95%CI: 0.63%– 2.71%; ANA: mean difference = 1.32%, 95%CI: 0.40%– 2.23%).

**Table 2:** Mean difference in product-matched band-specific FP rates for samples containing non-*Plasmodium* infectious agents or immunological factors and clean negative samples.

Sample type	Mean difference between FP rate on sample and FP rate on clean negative (n)		
	Pf band	Pan band	Pv band
Non-*Plasmodium* infectious agents			
Dengue	0.7% (218)	0.0% (113)	-0.2% (55)
Schistosomiasis	0.3% (184)	-0.1% (88)	-0.4% (50)
Leishmaniasis	1.7%* (218)	0.2% (113)	-0.2% (55)
Chagas	0.9% (218)	0.1% (113)	-0.3% (55)
Immunological factors			
RF	10.7%** (218)	16.5%** (113)	6.6%** (55)
ANA	1.3%* (218)	0.7% (113)	0.9%* (55)
HAMA	20.7%** (182)	18.1%** (90)	9.4%** (54)
RPR	0.2% (218)	-0.3% (113)	0.3% (55)

* 0.05 < p-value < 0.001.

** p-value ≤ 0.001.

In contrast, large differences were seen between the paired FP rates against clean negative samples and HAMA (*P*<0.001, Table 2); on average, HAMA samples had a FP rate 20.69% (95%CI: 17.08%– 24.30%) higher than the clean negative FP rate for the same product. A similar result was obtained for RF (*P*<0.001), with RF samples having a FP rate that was, on average, 10.73% (95%CI: 8.21%– 13.25%) higher than the product-specific clean negative FP rate. The FP rates for RF and HAMA were significantly correlated (correlation coefficient = 0.576, *P*<0.001).

### False positive (FP) results on pan *Plasmodium* test bands

The pan *Plasmodium* test bands detected either pLDH (86.7%, 98/113) and/or aldolase (15.96%, 18/113). Between 42% and 94% of RDTs had no FP against individual infectious agents or immunological factors on the pan test band (Fig 1). More than one quarter (26.5%, 30/113) had no FP against the entire panel of infectious agents and immunological factors. There were no significant differences between the paired FP rates on the pan test band against clean negative samples and samples containing dengue, Schistosomiasis, Leishmaniosis, Chagas, ANA or RPR (*P*>0.1, Table 2).

Similar distributions of FP rates were observed for HAMA and RF (Fig 1), with both types of samples having significantly higher FP rates than their product-matched clean negative FP rates (*P*<0.001). On average, HAMA samples had a FP rate that was 18.10% (95%CI: 13.28%– 22.92%) higher than the clean negative FP rate, while the FP rate for RF samples was 16.49% (95%CI: 12.41%– 20.57%) higher (Table 2). The FP rates on the pan band for RF and HAMA were significantly correlated (correlation coefficient = 0.686, *P*<0.001).

### False positive results on *P*. *vivax* test bands

All *P*. *vivax-*specific test bands detected pLDH. The FP rates of *P*. *vivax*-specific test bands were typically low, with between 65% and 95% of RDTs having no FP against specific infectious agents or immunological factors (Fig 1) and 50.9% (28/55) having no FP against any infectious agent or immunological factor in the panel. There were no significant differences between the paired FP rates on the Pv test band against clean negative samples and samples containing dengue, Schistosomiasis, Leishmaniasis, Chagas or RPR (*P*>0.05, Table 2). A statistically significant, but clinically irrelevant, difference was detected between the FP on clean negative samples and samples containing ANA (mean difference = 0.90%, 95%CI: 0.14% - 1.66%, *P* = 0.021).

Both HAMA and RF samples showed increased FP rates on the *P*. *vivax* test band compared to clean negative samples (*P*<0.001). HAMA samples had a FP rate that was, on average, 9.40% (95%CI: 4.91%– 13.89%) higher than the FP on clean negative samples for the same product. This value was 6.57% (95%CI: 2.15%– 10.99%) for RF. There was a significant correlation between the FP rates for RF and HAMA (correlation coefficient = 0.683, *P*<0.001).

### Correlations between different test bands for combination RDTs

The FP rates for each infectious agent and immunological factor were significantly correlated between test bands of combination RDTs (*P*≤0.002). For the Pf-pan combination RDTs, the Spearman Rank correlation coefficients for the eight types of infectious agents and immunological factors ranged from 0.456 (n = 97) for Schistosomiasis to 0.879 (n = 77) for HAMA. For the 50 Pf-Pv combination RDTs, the Spearman Rank correlation coefficients for the eight sample types ranged from 0.407 for Leishmaniasis to 0.815 for Chagas.

## Discussion

The aggregated results from six rounds of the WHO Malaria RDT Product Testing programme indicate that there are a substantial number of malaria RDTs which do not show any FP results against any of the non-*Plasmodium* infectious agents or immunological factors tested. Hence, it would appear that FP results against specific sample types is not a universal property of RDTs, but rather is related to the manufacturing process and/or specific antibody used in the RDT, which likely varies between products. The correlation between FP rates on different test bands of the same RDT further points to the importance of the manufacturing process in producing RDTs with high specificity.

It is notable that none of the non-*Plasmodium* infectious agents tested had clinically-relevant increases in FP rates compared to the product-matched FP rates on clean negative samples, suggesting that cross-reactivity against these specific agents is likely not a common occurrence in regions where these infectious diseases co-exist. This study did not include HAT samples, but consideration should be given to adding such samples to future RDT evaluations based on the high FP rates reported for some RDTs [2]. Data reported by Gillet et al [2] indicate the median difference in specificity of 10 RDTs tested against HAT samples was 12.6% lower than matched control samples, with values ranging from 1.2–68.2% for individual RDTs.

When an RDT did produce FP results, these rates were highest for samples containing RF and HAMA. Although the RF result is based on only 16–24 tests for each product, the finding aligns with published literature for a small number of RDTs [7–10]. The concentrations of RF in the samples analysed in this study ranged between 104 IU/ml and 1063 IU/ml. These concentrations are within the ranges reported from patients residing in non-endemic areas where FP RDT results were reported [7, 9]. Gillet et al [2] reported 4.3% of their samples from DR Congo had RF but did not find increased FP rates on 10 malaria RDTs against these RF-positive samples. However the maximum RF concentration in their samples was 47 IU/ml, considerably lower than in other studies. Since RF seroprevalence is associated with viral, parasitic and chronic bacterial infections [8, 14, 15], the higher FP rates may be a concern in many malaria endemic countries, especially countries with high prevalence of hepatitis B and C [7, 15].

The number of tests against HAMA was small with only 12 tests per product in Rounds 2–6. HAMA concentrations in these samples ranged from 600ng/ml to 846 ng/ml. Given the high FP rates demonstrated against HAMA, and the lack of previous reports of reactivity with malaria RDTs, further studies investigating this immunological factor are warranted. The clinical impact of these high FP rates is difficult to determine as the reported prevalence of patients with HAMA varies widely from <1% to 80%, with HAMA detection dependent on the type of assay and significant intermethod and interlaboratory differences in HAMA results on standard specimen panels [16]. It is also not clear where the HAMA concentrations used in this study fit in the spectrum of concentrations observed within HAMA-positive patients.

The technical specifications recently published by WHO for prequalification of malaria RDTs require the impact of both HAMA and RF to be assessed when determining analytical specificity [17]. However, until more malaria RDTs become prequalified under these technical specifications the current data suggests that practitioners may need to consider the possibility of false positive results for malaria in patients with conditions that stimulate HAMA or RF.

## Supporting information

S1 Table Products which did not return any false positive results against the non-*Plasmodium* infectious agents or immunological factors tested during rounds 1 to 6 of WHO product testing. (DOCX)
