# Supplementary material for: An assessment of false positive rates for malaria rapid diagnostic tests caused by non-Plasmodium infectious agents and immunological factors
Source: PLoS One. 2018 May 14;13(5):e0197395. doi: 10.1371/journal.pone.0197395 (PMC5951549; doi:10.1371/journal.pone.0197395)
Supplement: S1 Table — (DOCX) [file pone.0197395.s001.docx]

**S1 Table. Products which did not return any false positive results against the non-*Plasmodium* infectious agents or immunological factors tested during rounds 1 to 6 of WHO product testing.**

| Product | Catalogue Number | Manufacturer | Product Testing Round | On 2017 WHO procurement list* |
| --- | --- | --- | --- | --- |
| ***Pf-only products*** |  |  |  |  |
| ABON™ Malaria P.f. Rapid Test Device (Whole Blood) | IMA-402 | ABON Biopharm (Hangzhou) Co. Ltd | 4 | No |
| Clearview® Malaria P.f. | VB01 | Vision Biotech (Pty) Ltd | 3 | No |
| Core™ Malaria Pf | MAL-190020 | Core Diagnostics | 3 | No |
| First Response® Malaria Ag *P. falciparum* (HRP2) Card Test | I13FRC | Premier Medical Corporation | 5 | Yes |
| First Response® Malaria Antigen *P. falciparum* (HRP2) Card Test | PI13FRC | Premier Medical Corporation Ltd. | 6 | Yes |
| FirstSign™ Malaria Pf | 2100CB-25 | Unimed International Inc. | 4 | Yes |
| HiSens Malaria Ag Pf HRP2 Card | HR3023 | HBI Co., Ltd. | 2 | No |
| ICT Diagnostics Malaria P.f. | ML01 | ICT INTERNATIONAL | 3 | No |
| Malaria Rapid Pf | VB01 | Vision Biotech (Pty) Ltd. | 1 | No |
| One Step Malaria P.F Test (Cassette) | 522352 | Blue Cross Bio-Medical (Beijing) Co., Ltd. | 4 | Yes |
| OnSite Malaria Pf Ag Rapid Test | R0114C | CTK Biotech, Inc. | 6 | Yes |
| Paracheck® Pf Device- Rapid test for *P. falciparum* Malaria Ver. 3 | 30301025 | Orchid Biomedical Systems | 3 | No |
| Paracheck® Pf-Rapid Test for *P.falciparum* Malaria Device | 302030025 | Orchid Biomedical Systems | 4 | Yes |
| Parahit-f TEST DEVICE FOR FALCIPARUM MALARIA | 25975 | Span Diagnostics Ltd. | 1 | No |
| RapiGEN BIOCREDIT Malaria Ag Pf (HRPII) | C10RHA25 | RapiGEN Inc. | 6 | Yes |
| SD BIOLINE Malaria Ag P.f. (HRP2/pLDH) | 05FK90 | Standard Diagnostics Inc. | 3** | Yes |
| ***Pf-pan products*** |  |  |  |  |
| ABON Malaria Pan/P.f. Rapid Test Device | IMA-B402 | ABON Biopharm (Hangzhou) Co. Ltd. | 3 | No |
| Advantage Malaria Pan + Pf Card | IR231025 | J. Mitra & Co. Pvt. Ltd. | 5 | Yes |
| Advantage Mal Card | IR221025 | J. Mitra & Co. Pvt. Ltd. | 5 | No |
| First Response® Malaria Ag. pLDH/HRP2 Combo Card Test | I16FRC | Premier Medical Corporation | 5 | No |
| HiSens Malaria Ag P.f/P.v Card | HR2823 | HBI Co., Ltd. | 2 | No |
| HiSens Malaria Ag Pf/Pv (HRP2/pLDH) Card | HR2923 | HBI Co., Ltd. | 2 | No |
| Is It… Malaria Pf/Pv Device | AL030 | Medsource Ozone Biomedicals | 6 | Yes |
| NanoSign Malaria Pf/Pv Ag | RMAD10 | Bioland, Ltd | 3 | No |
| SD BIOLINE Malaria Ag | 05FK40 | Standard Diagnostics Inc. | 3 | No |
| SD BIOLINE Malaria Ag P.f/Pan | 05FK60 | Standard Diagnostics Inc. | 3** | No |
| ***Pf-Pv products*** |  |  |  |  |
| Advanced Quality™ One Step Malaria (Pf/Pv) Tri-line Test (whole blood) | ITP11003 TC40 | InTec Products, Inc. | 6 | No |
| Advantage Malaria Card | IR211025 | J. Mitra & Co. Pvt. Ltd. | 3 | No |
| CareStart™ Malaria HRP2/PLDH (Pf/Pv) COMBO | G0161 | Access Bio, Inc. | 2 | No |
| Core™ Malaria Pv/Pf | MAL-190022 | Core Diagnostics | 3 | No |
| First Response® Malaria Ag Pf/Pv Card Test | PI19FRC | Premier Medical Corporation Ltd. | 6 | No |
| HiSens Malaria Ag P.f/VOM Combo Card | HR3323 | HBI Co., Ltd. | 4 | Yes |
| One Step Malaria P.f/P.v Whole Blood Test | W056-C | Guangzhou Wondfo Biotech Co. Ltd. | 5** | No |
| OnSite Malaria Pf/Pv Ag Rapid Test | R0112C | CTK Biotech, Inc. | 6 | No |
| RapiGEN BIOCREDIT Malaria Ag Pf/Pv (HRPII/pLDH) | C40RHA25 | RapiGEN Inc. | 6 | Yes |
| ***Pan-only products*** |  |  |  |  |
| Advantage Pan Malaria Card | IR013025 | J. Mitra & Co. Pvt. Ltd. | 1 | No |
| Advantage Pan Malaria Card | IR013025 | J. Mitra & Co. Pvt. Ltd. | 5 | Yes |
| diagnosticks MALARIA (Pan) Cassette | MPNWBC1007.3 | SSA Diagnostics & Biotech Systems | 3 | No |
| First Response Malaria Ag pLDH | I12FRC30 | Premier Medical Corporation Ltd. | 2 | No |
| Parabank™ Device - Rapid test for Malaria Pan | 50301025 | Zephyr Biomedical Systems | 3 | No |

* Tests will not be on WHO procurement list if they do not meet the procurement criteria or were last tested in Rounds 1, 2 or 3 due to the requirement for resubmission every 5 years.

** Same product resubmitted to a later product testing round did return false positive results against the non-*Plasmodium* infectious agents or immunological factors. The 2017 WHO procurement list will reflect the characteristics of the most recent submission of the product.
